# Use of resuscitative endovascular balloon occlusion of the aorta (REBOA) for trauma and its performance in Japan over the past 18 years: a nationwide descriptive study

**DOI:** 10.1186/s13017-024-00548-5

**Published:** 2024-05-31

**Authors:** Hiromasa Hoshi, Akira Endo, Ryo Yamamoto, Kazuma Yamakawa, Keisuke Suzuki, Tomohiro Akutsu, Koji Morishita

**Affiliations:** 1 Department of Acute Critical Care Medicine, Tsuchiura Kyodo General Hospital, 4-1-1 Otsuno, Tsuchiura-shi, Ibaraki 300-0028 Japan; 2 Department of Acute Critical Care and Disaster Medicine, Graduate School of Medicine and Dental Sciences, Tokyo Medical and Dental University, Bunkyo, Tokyo Japan; 3 Department of Emergency and Critical Care Medicine, Keio University School of Medicine, Shinjuku, Tokyo Japan; 4 Department of Emergency and Critical Care Medicine, Osaka Medical and Pharmaceutical University, Takatsuki, Osaka Japan

**Keywords:** Resuscitative endovascular balloon occlusion of the aorta, Trauma, Hemorrhage, Aortic cross-clamping

## Abstract

**Background:**

Resuscitative endovascular balloon occlusion of the aorta (REBOA) has been used to control massive hemorrhages. Although there is no consensus on the efficacy of REBOA, it remains an option as a bridging therapy in non-trauma centers where trauma surgeons are not available. To better understand the current landscape of REBOA application, we examined changes in its usage, target population, and treatment outcomes in Japan, where immediate hemostasis procedures sometimes cannot be performed.

**Methods:**

This retrospective observational study used the Japan Trauma Data Bank data. All cases in which REBOA was performed between January 2004 and December 2021 were included. The primary outcome was the in-hospital mortality rate. We analyzed mortality trends over time according to the number of cases, number of centers, severity of injury, and overall and subgroup mortality associated with REBOA usage. We performed a logistic analysis of mortality trends over time, adjusting for probability of survival based on the trauma and injury severity score.

**Results:**

Overall, 2557 patients were treated with REBOA and were deemed eligible for inclusion. The median age of the participants was 55 years, and male patients constituted 65.3% of the study population. Blunt trauma accounted for approximately 93.0% of the cases. The number of cases and facilities that used REBOA increased until 2019. While the injury severity score and revised trauma score did not change throughout the observation period, the hospital mortality rate decreased from 91.3 to 50.9%. The REBOA group without severe head or spine injuries showed greater improvement in mortality than the all-patient group using REBOA and all-trauma patient group. The greatest improvement in mortality was observed in patients with systolic blood pressure ≥ 80 mmHg. The adjusted odds ratios for hospital mortality steadily declined, even after adjusting for the probability of survival.

**Conclusions:**

While there was no significant change in patient severity, mortality of patients treated with REBOA decreased over time. Further research is required to determine the reasons for these improvements in trauma care.

## Background

Hemorrhage is one of the primary causes of mortality in trauma cases and accounts for an estimated 30–40% of traumatic deaths [1]. Resuscitation of massive hemorrhages often requires massive transfusions and fluids, and rapid hemorrhage control is essential. Traditionally, aortic cross-clamping (ACC) has been used to temporarily control massive hemorrhage. However, ACC adds further invasiveness to a patient who has already sustained severe trauma, and managing bleeding from the incision site poses additional challenges [2]. Resuscitative endovascular balloon occlusion of the aorta (REBOA) has emerged as an alternative approach for controlling hemorrhages with minimal invasion [3]. However, its effectiveness is controversial. Some studies have shown that compared to ACC, REBOA has a better prognosis [2, 4], while others suggested that it is associated with a poorer prognosis [5–7]. Recently, a randomized trial conducted in major trauma centers in the United Kingdom suggested that REBOA increased the risk of death and prolonged the time to definitive hemostasis [7]. However, REBOA remains an option as a bridging therapy in non-trauma centers where trauma surgeons are not always available.

The situation surrounding REBOA is progressing without sufficient evidence. Off-the-job training courses for REBOA have been conducted worldwide, and devices have been improved, including the release of a narrower-diameter access route [8, 9], based on theoretical benefits. However, the validation of these efforts is insufficient. To better understand the current situation surrounding REBOA, we examined changes in its usage, target population, and treatment outcomes in Japan, where immediate hemostasis procedures sometimes cannot be performed.

## Methods

### Study design and settings

This retrospective observational study used data from the Japan Trauma Data Bank. The study was conducted from January 2004 to December 2021. The JTDB is required to register all severe trauma cases with AIS 3 or higher injuries and was established by the Japanese Association for Acute Medicine and Japanese Association for The Surgery of Trauma to understand the current status and improve the quality of trauma care, akin to the Trauma Quality Improvement Program in the United States. By the end of December 2021, the JTDB included 303 facilities providing trauma care in Japan, of which 95% were government-certified tertiary care centers.

In Japan, trauma patients are usually transported by ambulance staffed with paramedics, although physician-staffed ground or air ambulance is dispatched to the field in some cases. However, REBOA is rarely performed in pre-hospital settings and is often performed after arrival at the emergency department. Moreover, blood transfusion is rarely performed in pre-hospital settings.

This study complied with the principles of the 1964 Declaration of Helsinki and its amendments and was approved by the Ethics Committee of Tsuchiura Kyodo General Hospital (approval number: 2022FY10). The requirement for informed consent from each patient was waived because of the study’s retrospective nature. We used the opt-out method, which provides opportunities to refuse to participate in the study through online information disclosure in our hospital. The study was conducted in accordance with the Strengthening the Reporting of Observational Studies in Epidemiology (STROBE) reporting statement.

### Study participants

The study included all cases for which REBOA was used between 2004 and 2021.

### Measurements

We collected the following patient information from the JTDB: age; sex; year of injury; trauma classification (blunt or penetrating); pre-hospital vital signs [systolic blood pressure (SBP), heart rate (HR), respiratory rate (RR), and percutaneous oxygen saturation (SpO2)]; Glasgow coma scale (GCS) score; time from emergency medical service dispatch to emergency department (ED) arrival; vital signs at ED arrival [SBP, HR, RR, and SpO2, body temperature, and GCS, abbreviated injury scale (AIS) score for each region, injury severity score (ISS), revised trauma score (RTS), status at hospital discharge (survival or death)]; lactic acid level; focused assessment with sonography in trauma (FAST) results; number of REBOA cases, cases of cardiac arrest on arrival, and ACC cases; and probability of survival based on the trauma and injury severity score (TRISS-Ps).

#### Definitions and outcomes

The AIS was calculated based on AIS 98 until 2018 and AIS 2008 after 2019 in accordance with the change in JTDB registration rule. Data for pre-hospital GCS score, SpO_2_, and lactic acid levels on ED arrival were only available after 2019 in the JTDB. Cardiac arrest was characterized by a recorded SBP of 0 mmHg based on the registration instructions of the JTDB. The primary outcome was survival at hospital discharge.

### Statistical analysis

The trend of the number, characteristics, and outcomes of patients treated with REBOA and the number of facilities where REBOA was used, according to admission year, was described. Trends in hospital mortality were also compared among specific subgroups, including all patients with trauma, patients treated with REBOA, patients treated with REBOA without severe head or spine injuries defined by AIS ≥ 3, patients treated with REBOA with shock upon hospital arrival (SBP < 80 mmHg), and patients treated with REBOA without shock upon hospital arrival.

Patient characteristics were described using median and interquartile range (IQR) for continuous variables and number and percentage (%) for categorical variables. The chi-square test was used with a significance level of 0.05 to test the association of variables. We conducted a single regression analysis to show the changes in annual mortality rates across some subgroups: all patients with trauma in the database, all cases in which REBOA was used, the REBOA group without severe head or spine injury of AIS ≥ 3, cases with SBP < 80 mmHg among patients for whom REBOA was used, and patients with SBP ≥ 80 mmHg among patients for whom REBOA was used. We conducted a logistic regression analysis for the annual mortality rate adjusted by TRISS-Ps. All statistical analyses were performed using R software version 4.3.1 (R Foundation for Statistical Computing, Vienna, Austria).

## Results

A total of 427,561 patients were registered with the JTDB between 2004 and 2021. Of them, 2557 patients were treated with REBOA and were deemed eligible for inclusion. Table 1 presents the baseline patient characteristics. Overall, the median age of the participants was 55 years, with male patients constituting 65.3% of the study population. Blunt trauma accounted for approximately 93.0% of the cases. The median transport time was 37 min. The median SBP at ED arrival was 70 mm Hg. The thoracic region had the highest median AIS score. FAST-positive cases accounted for 45.9% of the cases. The median ISS and RTS values were 34.0 and 4.7, respectively. The median TRISS-Ps was 0.35. The overall mortality rate was 59.7%, and 16.9% of the patients experienced cardiac arrest upon arrival. ACC was performed in 16.7% of the cases. The number of REBOA cases was 24 in 2004, which increased to 262 in 2019. The number of REBOA cases declined from 2020 to 167 by 2021. The in-hospital mortality rate decreased from 91.3 to 50.9% during the observation period.

**Table 1:** Characteristics of patients over 18 years

Variables	Overall	2004	2005	2006	2007	2008	2009	2010	2011	2012	2013	2014	2015	2016	2017	2018	2019	2020	2021
n	2557	24	34	32	82	104	121	121	129	114	139	167	203	219	220	201	262	211	167
Age (year), median (IQR)	55 (36, 72)	60 (33, 69)	58 (39, 76)	56 (42, 70)	51 (28, 72)	57 (35, 70)	51 (30, 69)	52 (33, 71)	52 (36, 70)	57 (38, 72)	50 (35, 71)	56 (35, 71)	58 (38, 70)	54 (36, 72)	57 (41, 74)	53 (37, 70)	57 (39, 74)	59 (41, 74)	59 (40, 74)
Male sex, n (%)	1669 (65.3)	19 (79.2)	25 (73.5)	20 (62.5)	54 (65.9)	67 (64.4)	79 (65.3)	76 (62.8)	84 (65.1)	74 (64.9)	84 (60.4)	120 (71.9)	133 (65.5)	145 (66.2)	144 (65.5)	115 (57.2)	159 (60.7)	147 (69.7)	117 (70.1)
Type of trauma, n (%)																			
Blunt	2378 (93.0)	22 (91.7)	33 (97.1)	29 (90.6)	76 (92.7)	99 (95.2)	116 (95.9)	114 (94.2)	118 (91.5)	106 (93.0)	126 (90.6)	159 (95.2)	187 (92.1)	199 (90.9)	205 (93.2)	189 (94.0)	238 (90.8)	201 (95.3)	154 (92.2)
Penetrating	149 (5.8)	1 (4.2)	1 (2.9)	2 (6.2)	5 (6.1)	4 (3.8)	4 (3.3)	7 (5.8)	8 (6.2)	7 (6.1)	11 (7.9)	7 (4.2)	13 (6.4)	12 (5.5)	12 (5.5)	10 (5.0)	22 (8.4)	10 (4.7)	13 (7.8)
From EMS dispatch to ED arrival, median (IQR)	37 (27, 55)	40 (25, 57)	38 (24, 47)	32 (22, 44)	38 (27, 62)	32 (24, 44)	32 (24, 47)	34 (26, 49)	35 (26, 52)	35 (26, 58)	38 (29, 60)	38 (27, 59)	40 (28, 55)	38 (28, 60)	41 (30, 61)	37 (26, 55)	35 (27, 50)	39 (28, 57)	38 (28, 53)
Pre-hospital vital signs																			
Systolic blood pressure (mmHg), median (IQR)	98 (79, 125)	84 (74, 124)	84 (70, 100)	103 (80, 119)	100 (82, 130)	98 (80, 120)	93 (80, 122)	100 (76, 122)	96 (76, 117)	92 (74, 127)	100 (80, 116)	110 (82, 142)	109 (82, 136)	96 (80, 116)	102 (80, 128)	100 (77, 125)	90 (73, 119)	96 (77, 129)	94 (75, 120)
Heart rate (/min), median (IQR)	100 (78, 120)	77 (57, 98)	105 (66, 120)	98 (75, 116)	83 (71, 110)	95 (80, 119)	90 (75, 120)	104 (84, 126)	100 (85, 120)	102 (80, 121)	100 (78, 126)	102 (87, 122)	96 (78, 120)	100 (78, 127)	100 (75, 120)	102 (80, 127)	102 (77, 120)	102 (79, 123)	101 (76, 120)
Respiratory rate (/min), median (IQR)	24 (18, 30)	19 (13, 24)	24 (9, 30)	24 (18, 30)	24 (18, 30)	24 (20, 30)	24 (20, 30)	24 (18, 30)	28 (24, 30)	24 (18, 30)	24 (18, 30)	24 (20, 30)	24 (20, 30)	24 (20, 30)	24 (18, 30)	24 (20, 30)	24 (18, 30)	24 (18, 30)	24 (18, 30)
Glasgow Coma Scale *	8 (3, 14)	None	None	None	None	None	None	None	None	None	None	None	None	None	None	None	9 (3, 14)	7 (3, 14)	8 (3, 14)
Saturation of percutaneous oxygen (%) *	95 (86, 99)	None	None	None	None	None	None	None	None	None	None	None	None	None	None	None	94 (85, 99)	95 (86, 99)	96 (86, 99)
Vital signs at ED arrival																			
Systolic blood pressure (mmHg), median (IQR)	70 (40, 102)	50 (0, 81)	40 (0, 67)	40 (30, 98)	75 (40, 105)	73 (40, 90)	61 (40, 87)	80 (40, 115)	77 (40, 101)	70 (40, 101)	64 (40, 95)	70 (40, 98)	79 (40, 111)	65 (40, 102)	74 (40, 98)	66 (40, 96)	75 (40, 110)	71 (40, 112)	73 (40, 105)
Heart rate (/min), median (IQR)	100 (71, 124)	78 (0, 109)	96 (0, 113)	96 (61, 127)	101 (78, 120)	98 (73, 116)	96 (70, 123)	107 (80, 130)	97 (68, 120)	98 (63, 120)	95 (70, 122)	110 (80, 130)	108 (80, 129)	101 (74, 128)	100 (70, 124)	102 (70, 126)	104 (80, 126)	100 (69, 124)	100 (73, 123)
Respiratory rate (/min), median (IQR)	22 (12, 30)	12 (0, 25)	6 (0, 28)	21 (7, 28)	22 (13, 30)	24 (16, 30)	24 (10, 30)	25 (16, 32)	24 (10, 30)	24 (12, 30)	22 (11, 30)	23 (12, 30)	22 (16, 30)	20 (3, 30)	20 (12, 30)	23 (12, 30)	22 (13, 30)	23 (10, 28)	20 (12, 30)
Body temperature (℃)	35.8 (35.0, 36.4)	35.0 (34.3, 35.8)	35.0 (35.0, 35.2)	35.0 (34.6, 35.8)	35.6 (35.0, 36.2)	35.9 (35.0, 36.5)	35.5 (35.0, 36.1)	35.4 (34.9, 36.0)	35.6 (35.1, 36.2)	35.5 (34.8, 36.3)	35.9 (35.2, 36.4)	35.8 (35.2, 36.4)	35.7 (34.8, 36.3)	35.9 (35.0, 36.4)	35.7 (34.9, 36.4)	36.0 (35.1, 36.4)	35.9 (35.2, 36.4)	36.1 (35.2, 36.6)	36.0 (35.1, 36.5)
Glasgow Coma Scale	7 (3, 13)	7 (3, 12)	3 (3, 6)	4 (3, 9)	7 (3, 14)	9 (3, 14)	8 (3, 14)	8 (3, 13)	7 (3, 13)	8 (3, 13)	7 (3, 13)	7 (3, 13)	8 (3, 13)	6 (3, 13)	7 (3, 13)	6 (3, 13)	6 (3, 13)	6 (3, 13)	7 (3, 13)
Saturation of percutaneous oxygen, (%) *	98 (92, 100)	None	None	None	None	None	None	None	None	None	None	None	None	None	None	None	98 (93, 100)	98 (92, 100)	98 (94, 100)
Lactate (mmol/l) *	7.1 (3.9, 11.1)	None	None	None	None	None	None	None	None	None	None	None	None	None	None	None	6.4 (3.5, 10.0)	7.6 (4.7, 11.6)	7.2 (3.7, 11.4)
AIS †																			
Head	3 (1, 4)	4 (3, 4)	4 (3, 5)	3 (2, 5)	3 (3, 5)	4 (3, 5)	3 (3, 5)	4 (3, 4)	3 (3, 5)	3 (3, 5)	3 (3, 4)	3 (3, 5)	4 (3, 5)	3 (3, 4)	4 (3, 5)	4 (3, 5)	0 (0, 3)	0 (0, 3)	0 (0, 3)
Face	1 (0, 2)	3 (1, 4)	2 (2, 2)	2 (1, 2)	1 (1, 2)	1 (1, 2)	2 (1, 2)	1 (1, 2)	1 (1, 2)	2 (1, 2)	2 (1, 2)	2 (1, 2)	2 (1, 2)	2 (1, 2)	2 (1, 2)	2 (1, 2)	0 (0, 0)	0 (0, 0)	0 (0, 0)
Neck	0 (0, 0)	NA	NA	5 (5, 5)	1 (1, 1)	3 (3, 3)	2 (1, 2)	NA	3 (3, 3)	2 (1, 2)	5 (5, 5)	1 (1, 1)	3 (2, 3)	1 (1, 2)	3 (2, 3)	3 (3, 4)	0 (0, 0)	0 (0, 0)	0 (0, 0)
Thorax	4 (3, 4)	4 (3, 5)	4 (3, 5)	4 (4, 4)	4 (3, 4)	4 (3, 5)	4 (3, 4)	4 (3, 4)	4 (3, 5)	4 (3, 4)	4 (4, 5)	4 (3, 5)	4 (3, 5)	4 (3, 5)	4 (3, 5)	4 (3, 4)	3 (2, 4)	3 (0, 4)	3 (0, 3)
Abdomen	3 (2, 4)	4 (4, 4)	4 (3, 4)	4 (3, 5)	3 (3, 4)	4 (3, 4)	4 (3, 4)	3 (3, 4)	3 (3, 4)	4 (3, 5)	4 (3, 4)	3 (3, 4)	3 (3, 4)	3 (3, 4)	3 (3, 4)	3 (3, 4)	3 (0, 4)	2 (0, 4)	2 (0, 3)
Spine	2 (0, 2)	2 (2, 2)	2 (2, 3)	3 (2, 4)	3 (2, 6)	2 (2, 3)	2 (2, 2)	3 (2, 3)	2 (2, 3)	2 (2, 3)	2 (2, 3)	2 (2, 3)	2 (2, 3)	2 (2, 3)	2 (2, 3)	2 (2, 3)	0 (0, 2)	0 (0, 2)	0 (0, 2)
Upper extremity	2 (0, 2)	2 (2, 2)	2 (2, 2)	2 (2, 2)	2 (2, 2)	2 (2, 2)	2 (2, 3)	2 (2, 3)	2 (2, 3)	2 (2, 2)	2 (2, 3)	2 (2, 3)	2 (2, 2)	2 (2, 3)	2 (2, 3)	2 (2, 3)	0 (0, 2)	0 (0, 2)	0 (0, 2)
Lower extremity	3 (2, 5)	4 (3, 5)	5 (3, 5)	5 (2, 5)	4 (3, 5)	4 (3, 5)	4 (3, 5)	4 (3, 5)	4 (3, 5)	4 (3, 5)	4 (3, 5)	4 (3, 5)	3 (3, 5)	3 (3, 4)	4 (3, 5)	4 (3, 5)	2 (0, 4)	2 (0, 4)	3 (0, 4)
External	0 (0, 0)	NA (NA, NA)	1 (1, 1)	1 (1, 1)	1 (1, 1)	1 (1, 1)	1 (1, 1)	1 (1, 2)	1 (1, 1)	1 (1, 1)	1 (1, 1)	1 (1, 1)	1 (1, 1)	1 (1, 1)	1 (1, 1)	1 (1, 1)	0 (0, 0)	0 (0, 0)	0 (0, 0)
ISS †	34 (25, 45)	38 (27, 42)	36 (25, 57)	37 (25, 49)	32 (22, 43)	38 (25, 50)	34 (22, 45)	34 (25, 44)	34 (25, 45)	41 (25, 50)	36 (25, 50)	37 (26, 50)	36 (25, 48)	34 (25, 45)	36 (25, 48)	37 (26, 50)	29 (22, 41)	29 (23, 41)	33 (24, 41)
RTS	4.7 (1.9, 6.6)	3.4 (0.0, 6.4)	0.7 (0.0, 4.4)	3.6 (1.4, 5.8)	5.0 (2.8, 6.9)	5.0 (2.6, 6.8)	4.9 (2.0, 6.4)	5.4 (3.5, 6.8)	4.9 (2.6, 6.4)	4.5 (2.3, 6.4)	5.1 (1.5, 6.4)	4.5 (2.3, 6.4)	5.3 (3.1, 6.8)	4.1 (1.9, 6.6)	4.7 (2.3, 6.5)	4.5 (1.9, 6.4)	5.1 (2.6, 6.8)	4.7 (1.9, 6.4)	4.9 (2.1, 6.7)
Cardiac arrest at ED arrival, n (%)	432 (16.9)	7 (29.2)	13 (38.2)	8 (25.0)	12 (14.6)	14 (13.5)	24 (19.8)	14 (11.6)	23 (17.8)	23 (20.2)	26 (18.7)	24 (14.4)	23 (11.3)	47 (21.5)	39 (17.7)	35 (17.4)	35 (13.4)	35 (16.6)	29 (17.4)
FAST, n (%)																			
Positive	1174 (45.9)	9 (37.5)	19 (55.9)	15 (46.9)	46 (56.1)	61 (58.7)	78 (64.5)	74 (61.2)	61 (47.3)	45 (39.5)	63 (45.3)	88 (52.7)	88 (43.3)	100 (45.7)	94 (42.7)	79 (39.3)	108 (41.2)	79 (37.4)	65 (38.9)
Negative	1104 (43.2)	12 (50.0)	11 (32.4)	15 (46.9)	27 (32.9)	36 (34.6)	34 (28.1)	42 (34.7)	48 (37.2)	56 (49.1)	61 (43.9)	65 (38.9)	95 (46.8)	102 (46.6)	97 (44.1)	87 (43.3)	126 (48.1)	107 (50.7)	78 (46.7)
Not performed	157 (6.1)	3 (12.5)	1 (2.9)	2 (6.2)	4 (4.9)	3 (2.9)	6 (5.0)	2 (1.7)	12 (9.3)	6 (5.3)	6 (4.3)	9 (5.4)	9 (4.4)	7 (3.2)	18 (8.2)	23 (11.4)	17 (6.5)	14 (6.6)	15 (9.0)
Aortic cross-clamping, n (%)	426 (16.7)	1 (4.2)	0 (0.0)	2 (6.2)	3 (3.7)	4 (3.8)	12 (9.9)	21 (17.4)	15 (11.6)	15 (13.2)	24 (17.3)	35 (21.0)	32 (15.8)	46 (21.0)	37 (16.8)	40 (19.9)	48 (18.3)	48 (22.7)	42 (25.1)
Provability of survival (%) †	0.35 (0.05, 0.79)	0.20 (0.04, 0.68)	0.03 (0.01, 0.16)	0.16 (0.07, 0.44)	0.46 (0.06, 0.83)	0.36 (0.07, 0.71)	0.36 (0.06, 0.79)	0.49 (0.14, 0.81)	0.35 (0.05, 0.80)	0.23 (0.06, 0.59)	0.33 (0.04, 0.80)	0.23 (0.03, 0.78)	0.39 (0.08, 0.77)	0.24 (0.04, 0.75)	0.34 (0.05, 0.75)	0.30 (0.04, 0.74)	0.53 (0.08, 0.87)	0.36 (0.06, 0.85)	0.53 (0.06, 0.85)
Mortality, n (%)	1527 (59.7)	21 (87.5)	28 (82.4)	28 (87.5)	58 (70.7)	69 (66.3)	89 (73.6)	77 (63.6)	86 (66.7)	77 (67.5)	82 (59.0)	102 (61.1)	112 (55.2)	131 (59.8)	125 (56.8)	107 (53.2)	132 (50.4)	112 (53.1)	85 (50.9)

*Abbreviations* IQR, interquartile range; EMS, emergency medical service; ED, emergency department; AIS, abbreviated injury scale; ISS, injury severity score; RTS, revised trauma score; FAST, focused assessment with sonography in trauma

* Data on some prehospital vital signs (Glasgow coma scale, saturation of percutaneous oxygen) and saturation of percutaneous oxygen at ED arrival, and lactate level at ED arrival exist only after 2019

† AIS, ISS, and provability of survival were calculated based on AIS 98 until 2018 and AIS 2008 after 2019

**Fig. 1:**
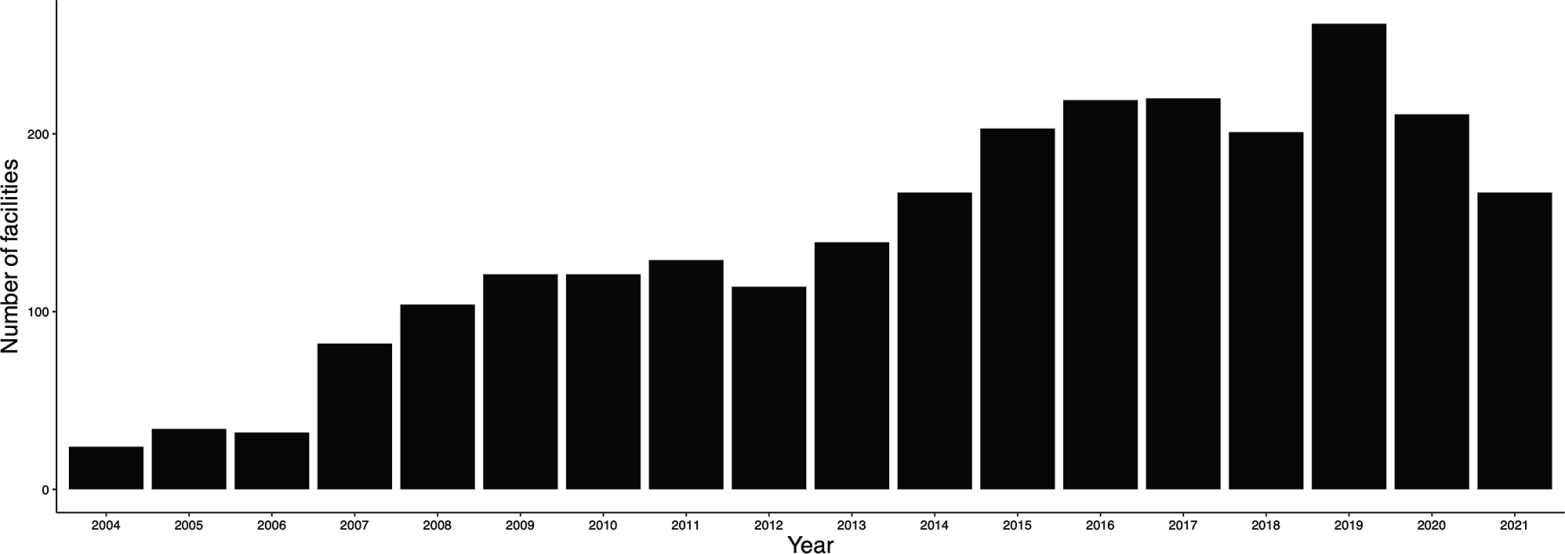
Number of facilities over time

**Fig. 2:**
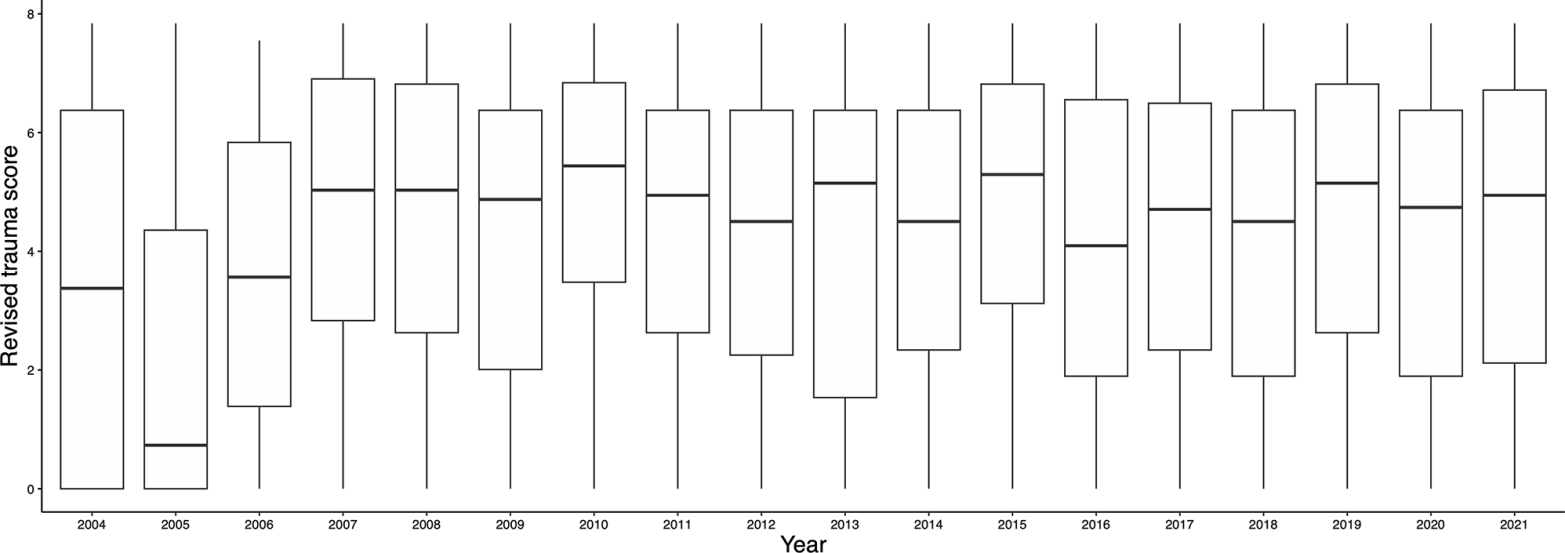
Changes in revised trauma score of the patients for whom REBOA was used. The box shows the interquartile range; the horizontal line is the median. *Abbreviations* REBOA, resuscitative endovascular balloon of aorta

**Fig. 3:**
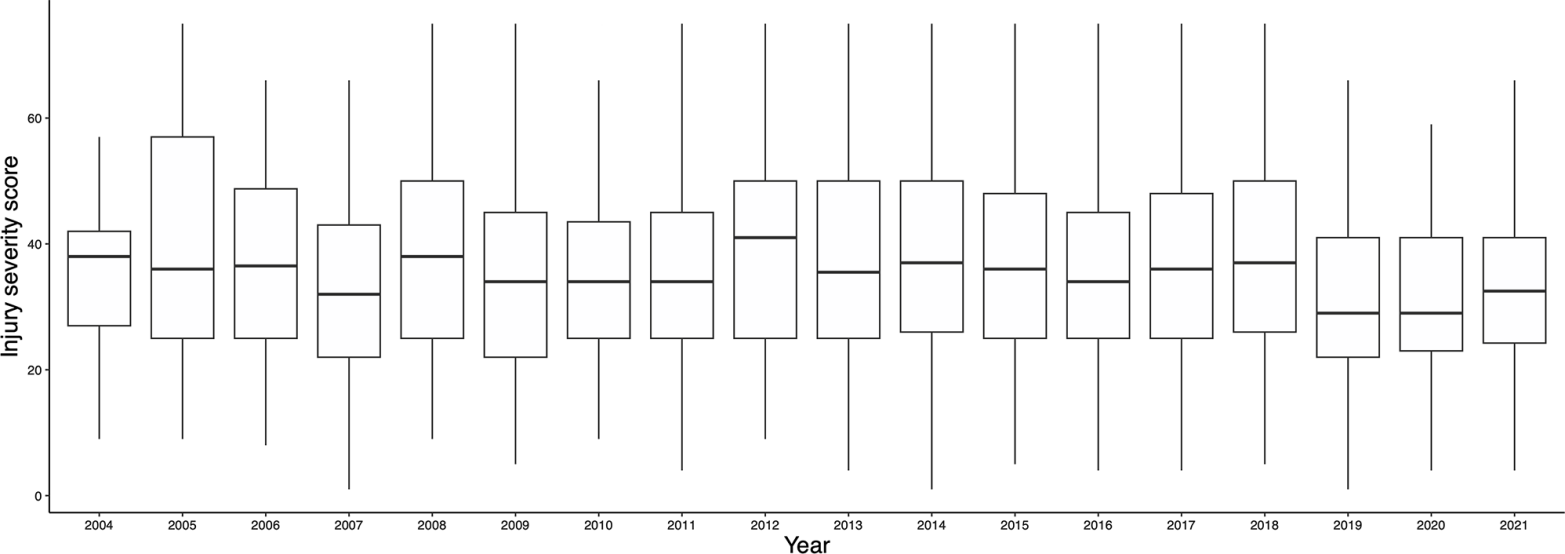
Changes in injury severity score of the patients for whom REBOA was used. The box shows the interquartile range; the horizontal line is the median. *Abbreviations* REBOA, resuscitative endovascular balloon of aorta

The number of facilities using REBOA in 2004 increased from 10 to 105 in 2019. The number of facilities using REBOA has decreased since 2020, reaching 62 by 2021 (Fig. 1). Although RTS changed substantially from 2004 to 2006, the median and IQR for both RTS (Fig. 2) and ISS (Fig. 3) were similar after these periods.

**Fig. 4:**
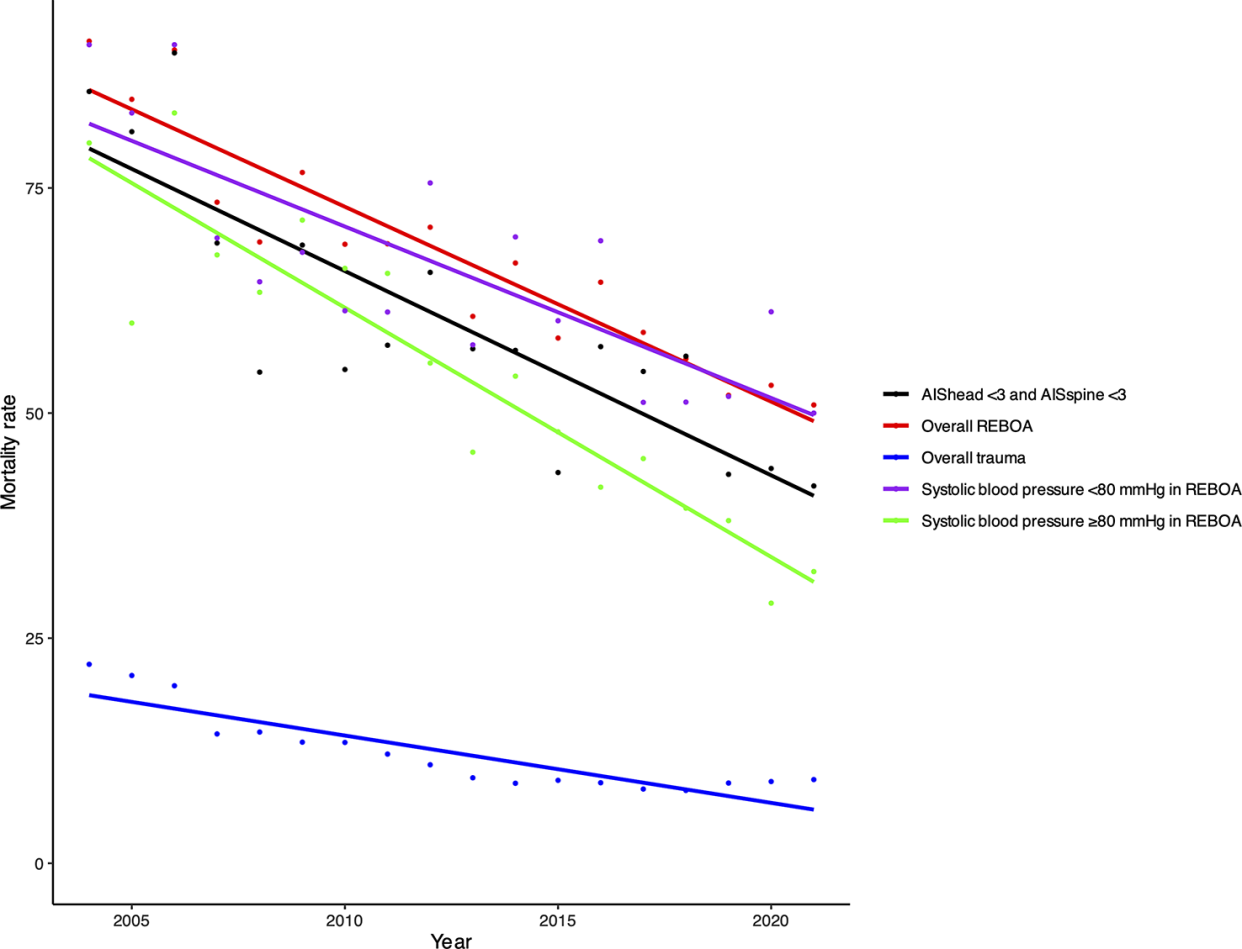
Mortality rate in subgroups and overall trauma over time. Single regression analysis was conducted in various subgroups to compare the trends in mortality rates. The black line shows the REBOA group without severe head or spine injury of AIS ≥ 3. The red lines show the cases in which REBOA was used. The blue line indicates trauma cases in the database. The purple line shows cases with SBP < 80 mmHg among those where REBOA was used. The green line shows cases with SBP ≥ 80 mmHg among cases where REBOA was used. *Abbreviations* REBOA, resuscitative endovascular balloon of the aorta; AIS, Abbreviated injury scale; SBP, systolic blood pressure

Univariate regression analysis was conducted in various subgroups to compare the trends in mortality rates (Fig. 4). The REBOA group without severe head or spine injury of AIS ≥ 3 had greater improvement in mortality than that of all-patient group using REBOA and all-trauma patient group. The greatest improvement in mortality was observed in patients with SBP ≥ 80 mmHg among all patients for whom REBOA was performed.

**Fig. 5:**
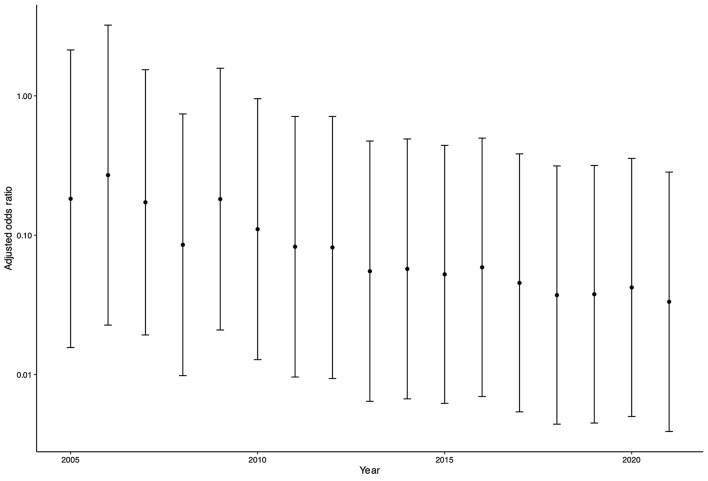
Trends in adjusted odds ratio of mortality. Logistic regression analysis was performed to determine the outcome of annual mortality adjusted by TRISS-PS. Adjusted odds ratios and 95% confidence intervals are shown

Logistic regression analysis for the outcome of annual mortality indicated steadily declined mortality even after adjusting for TRISS-Ps (Fig. 5).

## Discussion

A retrospective observational study analyzing data from the Japanese nationwide trauma registry over the past 18 years was conducted on 2,557 patients who underwent REBOA. A consistently decreasing trend in mortality was observed. The physiological and anatomical severities of the patients were similar over the years, and a reduction in mortality was observed, regardless of the severity of their conditions.

Several previous studies evaluated the mortality rate associated with REBOA, ranging from 34 to 70.8% [10–13]. Aoki et al. showed that the mortality rate of patients undergoing REBOA decreased between 2004 and 2015 [14]. Our study further enriched these results by adding 6 years of data, demonstrating changes over time in the number of cases in which REBOA was used, number of facilities using REBOA, and analysis of specific subgroups. The results of the present study revealed that mortality continued improving after 2016. The increase in the use of REBOA and the number of facilities implementing it until 2019 may be attributed to the growing recognition of the procedure. The introduction of hybrid emergency room system in Japan in 2011 partially could have contributed to this trend [15, 16].

Furthermore, 7 Fr narrow-diameter balloons were approved for clinical use in 2013. They were shown to reduce complications related to REBOA [17], including lower extremity ischemia. This technical progress in REBOA may have resulted in its increased number of REBOA uses. The decrease in the number of patients and facilities after 2020 could be attributed to the COVID-19 pandemic. Given that the RTS and ISS remained similar over the years, it was suggested that the indications for REBOA were consistent over 18 years.

While trauma-related mortality has decreased over the years [18], the mortality rate of patients treated with REBOA was further reduced compared to that of the all-trauma cohort [18]. There are several possible explanations for this. It is possible that the REBOA insertion technique has been partially improved by disseminating off-the-job REBOA training courses in Japan [8, 19]. Among the cases of REBOA, a significant reduction in mortality was observed in patients with an SBP of 80 mmHg or higher, indicating that REBOA had a particularly significant effect on improving outcomes in bleeding patients with relatively stable conditions. The UK-REBOA randomized clinical trial suggested that there may be more deaths in the REBOA group, which may have been influenced by the longer time to hemostasis in the REBOA group. Furthermore, the improvement in the mortality rate of patients without severe head or spine trauma was lower than that of patients treated with REBOA. These findings indicate that the progress in the hemostatic strategy, including damage control resuscitation, contributed to the favorable outcome of the patients who underwent REBOA.

This study has some limitations. Because this was retrospective registry-based study, some important information was missing, namely when, where, and how REBOA was performed. Since information on changes in vital signs after arrival at hospital was not available, the patients’ condition just before REBOA was unclear. The zone, extent, duration of the REBOA inflation, and the method used to confirm the placement of the REBOA catheter was unclear. The expertise level of the individual performing REBOA was unknown. Detailed information on the preparation and operation of hemostatic procedures, such as the availability of trauma surgeons and operating theaters at each facility, is unavailable in the JTDB.

## Conclusions

While there was no significant change in patient severity, mortality of patients treated with REBOA decreased over time. Further research is required to determine the reasons for these improvements in trauma care.

## Data Availability

The data that supported the findings of this study are available from the Japan Trauma Data Bank, but the availability of these data is restricted.

## Abbreviations

REBOA: Resuscitative endovascular balloon occlusion of the aorta
ACC: aortic cross-clamping
SBP: systolic blood pressure
HR: heart rate
RR: respiratory rate
SpO2: percutaneous oxygen saturation
GCS: Glasgow coma scale
ED: emergency department
AIS: abbreviated injury scale
ISS: injury severity score
RTS: revised trauma score
FAST: focused assessment with sonography in trauma
TRISS-Ps: trauma and injury severity score
IQR: interquartile range; percentage, %
